# Autogenous structural bone graft reconstruction of ≥ 10-mm-deep uncontained medial proximal tibial defects in primary total knee arthroplasty

**DOI:** 10.1186/s10195-024-00762-6

**Published:** 2024-04-23

**Authors:** Ahmed Abdel-Monem Dewidar, Mohamed Kamal Mesregah, Mustafa Mohamed Mesriga, Ahmed Mohamed El-Behiry

**Affiliations:** Department of Orthopaedic Surgery, Menoufia University Faculty of Medicine, Shebin-El-Kom, Menoufia, Egypt

**Keywords:** Total knee arthroplasty, Primary TKA, Tibial defects, Uncontained defects, Autologous bone graft, Autograft

## Abstract

**Background:**

Management of uncontained medial proximal tibial defects during primary total knee arthroplasty (TKA) can be challenging, especially for defects ≥ 10 mm in depth. This study sought to assess the outcomes of autogenous structural bone grafts to address these defects.

**Materials and methods:**

In this prospective study, patients with uncontained medial proximal tibial defects ≥ 10 mm in depth undergoing TKA were managed by autogenous structural bone grafts fixed by screws and were followed up for at least 36 months. Patients were followed-up clinically with Knee Society Score (KSS) and Western Ontario and McMaster Universities Osteoarthritis Index (WOMAC). Additionally, radiological follow-up was done to assess bone graft union and implant stability.

**Results:**

The study included 48 patients with a mean age of 69.2 ± 4.5 years. The mean body mass index (BMI) was 31.4 ± 3.7 kg/m^2^. The mean defect depth was 17 ± 3.6 mm. With a mean follow-up period of 52.2 ± 12.3 months, the median KSS improved significantly from 30 preoperatively to 89, *P* < 0.001. The median WOMAC score reduced significantly from 85 preoperatively to 30.5, *P* < 0.001. The mean ROM increased significantly from 73 ± 12.4 preoperatively to 124 ± 8.4 degrees, *P* < 0.001. The mean graft union time was 4.9 ± 1 months. No significant complications were reported.

**Conclusions:**

Autogenous bone graft reconstruction is a safe and effective method of addressing uncontained medial proximal tibial defects in primary TKA.

**Level of evidence:**

Level IV.

## Introduction

Knee osteoarthritis (OA) is a common age-related disorder that may lead to significant pain, stiffness, and reduced knee function, especially in the advanced degrees [1, 2].

Total knee arthroplasty (TKA) is a widely employed intervention for treating end-stage knee OA, aiming to establish a knee that mimics the natural function and kinematics of the native knee [3, 4]. Adequate preoperative planning, achieving precise implant positioning, correcting the limb alignment, restoring the joint line, and ensuring gap balancing are imperative for the success of the TKA procedure [5, 6].

Patients experiencing advanced knee OA may present with a significant varus deformity, often accompanied by bone defects, especially in the posteromedial aspect of the tibia due to degenerative erosions. These uncontained defects do not provide peripheral support for the implant components [7, 8].

Addressing these bone defects is of paramount significance for the success of TKA [9, 10]. If not appropriately addressed, these defects can compromise the bone–implant interface, leading to implant components loosening and malalignment and a greater likelihood of requiring revision surgery [11]. After resection of the tibial plateau in TKA surgery, any defect exceeding 10 mm in its largest diameter typically requires reconstruction [9].

Bone defects can be managed in several ways depending on the defect size and its containment status after the tibial bone cut [7]. Bone cement is used to fill defects less than 5 mm deep. Additionally, the approach of increased tibial resection with the use of a thicker polyethylene insert may be applied for defects of less than 10 mm [12].

If bone deficiencies exceed 10 mm, it is advisable not to cut the tibia to the level of the deficit, as distal tibial resection weakens osseous support, leading to a decreased area of support and increased loading [13, 14]. Therefore, reconstruction using allograft, autograft, metal augments, cones, and metaphyseal sleeves should be employed [9, 10, 15, 16].

The use of metal augments to address uncontained defects ≥ 10 mm deep has been described with good functional outcomes [16]. However, this technique raises medical expenses, necessitates additional bone cutting involving the cortical bone, and can complicate future revision surgeries [15, 16].

Few knee arthroplasty studies have evaluated the use of autogenous or allogenous bone grafts in dealing with uncontained defects exceeding 10 mm in depth in the medial proximal tibia in primary cases [9, 13].

Therefore, this study was conducted to assess the functional and radiological results of structural autograft bone reconstruction of medial proximal tibial defects ≥ 10 mm deep in primary TKA and to investigate the preoperative factors affecting the results.

## Material and methods

This study was a prospective study of patients with uncontained medial proximal tibial defects undergoing primary TKA between March 2015 and March 2020. Written consent from participants was obtained, along with approval from the institutional review board (IRB). Surgeries were performed by a single surgeon (A.A.D.).

Inclusion criteria were patients with Kellgren and Lawrence grade 4 (KL4) OA with varus deformity and uncontained medial proximal tibial defects ≥ 10 mm deep after the proximal tibial cut. Patients should have completed at least 36 months of follow-up to be included.

Patients with previous knee surgery, valgus OA, rheumatoid arthritis, infection, osteonecrosis, defects after tumor resection, contained defects, associated significant distal femur defects, Charcot knee, defects less than 10 mm depth, or use of metal augmentations were excluded.

### Preoperative assessment

History taking was done, including analysis of symptoms of pain, stiffness, instability, up-stairing, down-stairing, gait, and rising from chair. The body mass index (BMI) was calculated, and patients were classified based on the World Health Organization (WHO) classification [17] as underweight (BMI < 18.5 kg/m^2^), healthy (18.5–24.9 kg/m^2^), overweight (25–29.9 kg/m^2^), and obesity class I (30–34.9 kg/m^2^), class II (35–39.9 kg/m^2^) or class III (≥ 40 kg/m^2^).

Complete limb examination was done with assessment of the varus deformity regarding the degree, whether correctable or fixed, and the associated deformities (flexion or rotatory). Additionally, the mediolateral instability and the lateral ligament laxity were assessed. Active and passive ranges of motion and patellar tracking were also evaluated.

**Fig. 1:**
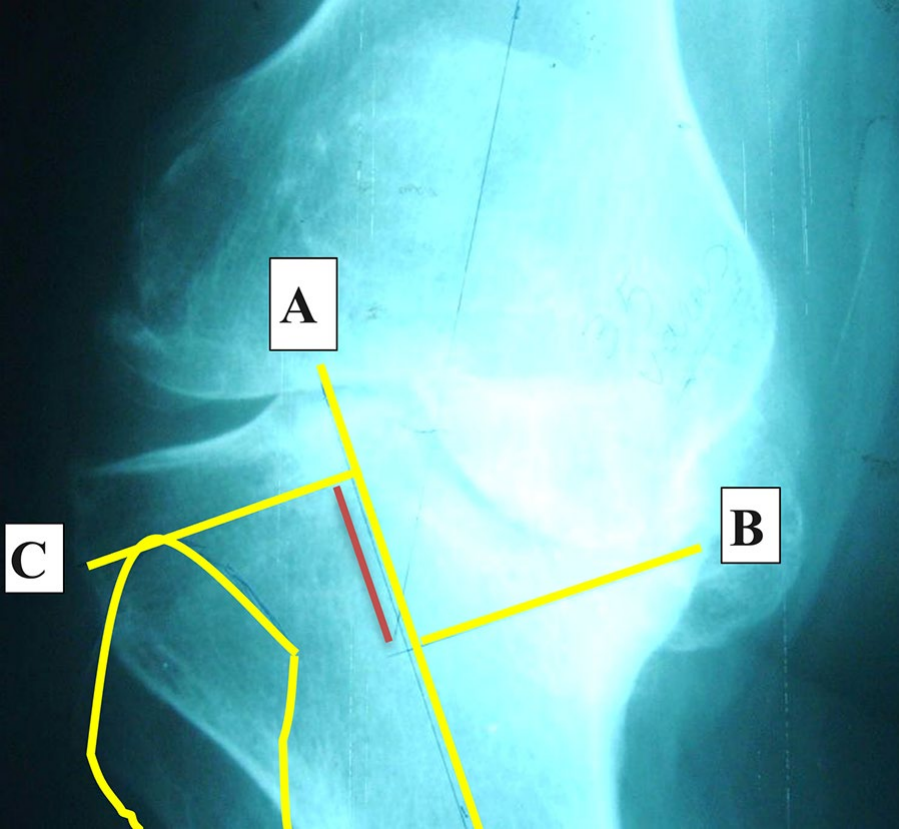
Knee plain X-ray AP view showing measurement of the medial tibial defect. Line **A** is through the anatomical axis of the tibia. Line **B** passes through the deepest point of the defect and is perpendicular to line **A**. Line **C** passes through the highest point of the head fibula and is perpendicular to line **A**, representing the expected resection line of the tibial plateau. The red line is the size of the defect

Knee anteroposterior (AP) and lateral standing plain X-rays were done to confirm the clinical diagnosis of advanced arthritis and assess the site, size, containment, shape, and slope of the bone defects. The depth of bone defect from the expected level of the proximal tibial cut was preliminary assessed in the preoperative AP X-ray, Fig. 1.

Long-film AP weight-bearing X-ray was done to evaluate the anatomical and mechanical axes and quantify the degree of varus deformity by measuring the anatomical femorotibial angle (aFTA).

Additional X-rays included a skyline view at 30 degrees flexion for evaluation of the patellar maltracking and stress views for evaluation of the coronal instability due to bone stock loss or ligamentous insufficiency.

### Surgical technique

Surgeries were done under combined epidural and spinal anesthesia. A standard medial parapatellar approach was used with traditional steps for preparation of the tibia and femur.

The proximal tibia was displaced anteriorly, and the tibial cut was done through the nondeficient lateral tibial plateau which was our reference in tibial cut by using a special stylus adjusted at 10 mm, with either intramedullary or extramedullary alignment guides. The tibial cut was done using an oscillating saw taking 10 mm from nonworn lateral tibial plateau leaving a defect in medial tibial plateau.

Dealing with the deficient medial tibial plateau was done using bone graft blocks from the proximal tibial or distal femoral bone cuts. Firstly, the concave and irregular surface of the defect was flattened by minimal bone removal using the oscillating saw with exposure of healthy bone to enhance further healing with the graft. The depth of the defect was evaluated and measured using a sterilized ruler.

**Fig. 2:**
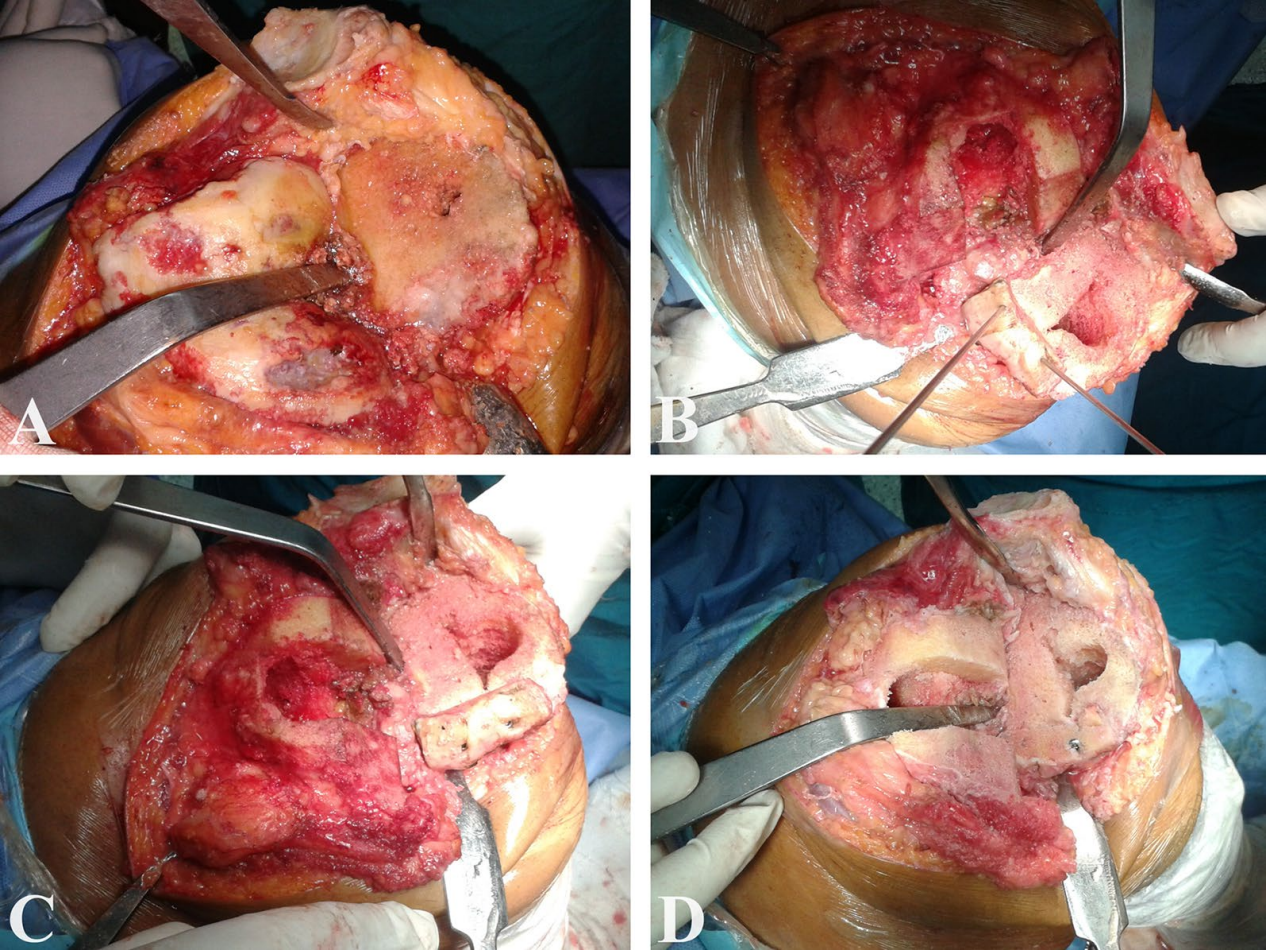
Intraoperative images of the bone graft preparation and insertion into the defect. **A** Evaluation of the defect after the proximal tibial cut. **B** Placement of the graft into the defect and provisional fixation by two K-wires. **C** Replacement of the two K-wires with two 3.5 mm partially threaded cancellous screws. **D** Removing the protruding part of the graft with an oscillating saw to create a flat upper tibial surface

The graft was fashioned using bony rongeur, placed over the flattened defect, and secured provisionally by Kirschner wires (K-wires). The K-wires were then replaced by 3.5 mm cancellous screws, making sure that they did not interfere with the tibial component keel or stem. The protruding part of the graft was then removed by an oscillating saw to create a flat upper tibial surface, Fig. 2.

Before cementation, the interface of the bone graft and the tibia was filled by impaction bone graft to avoid the extrusion of cement into this interface.

Trial components were inserted for assessment of size, prosthesis fitting, position, equality of bone gaps, and traditional restoration of neutral mechanical alignment was important as it had great effect on bone graft survival and prosthesis loosening. Tibial stem was used in all cases to protect the bone graft from stress.

The definitive prosthesis was inserted by the routine cementing technique. Posterior-stabilized (PS) TKA with a stemmed tibial component was used to unload the deficient metaphyseal bone. In cases with severe lateral collateral ligament laxity, Legacy Constrained Condylar Knee (LCCK) prosthesis (Zimmer-Biomet, Warsaw, Indiana, USA) was used.

Patelloplasty was done by removing all the osteophytes by the nibbler and denervation of the patella by applying cautery circumferentially around the patella (patellar circumcision).

Good hemostasis was achieved after the release of the tourniquet, followed by closure of the wound after application of a suction drain.

### Postoperative care and follow-up

Epidural postoperative analgesia was given in the ward using a continuous syringe pump system for sustained analgesia for 48 h postoperatively. Additionally, intravenous antibiotics were given for 48 h postoperatively. Static quadriceps and hamstring muscle strengthening exercises and straight leg raising exercises were commenced from day one, in addition to active and assisted flexion–extension range of motion (ROM) exercises. Weight-bearing was permitted without limitations.

**Fig. 3:**
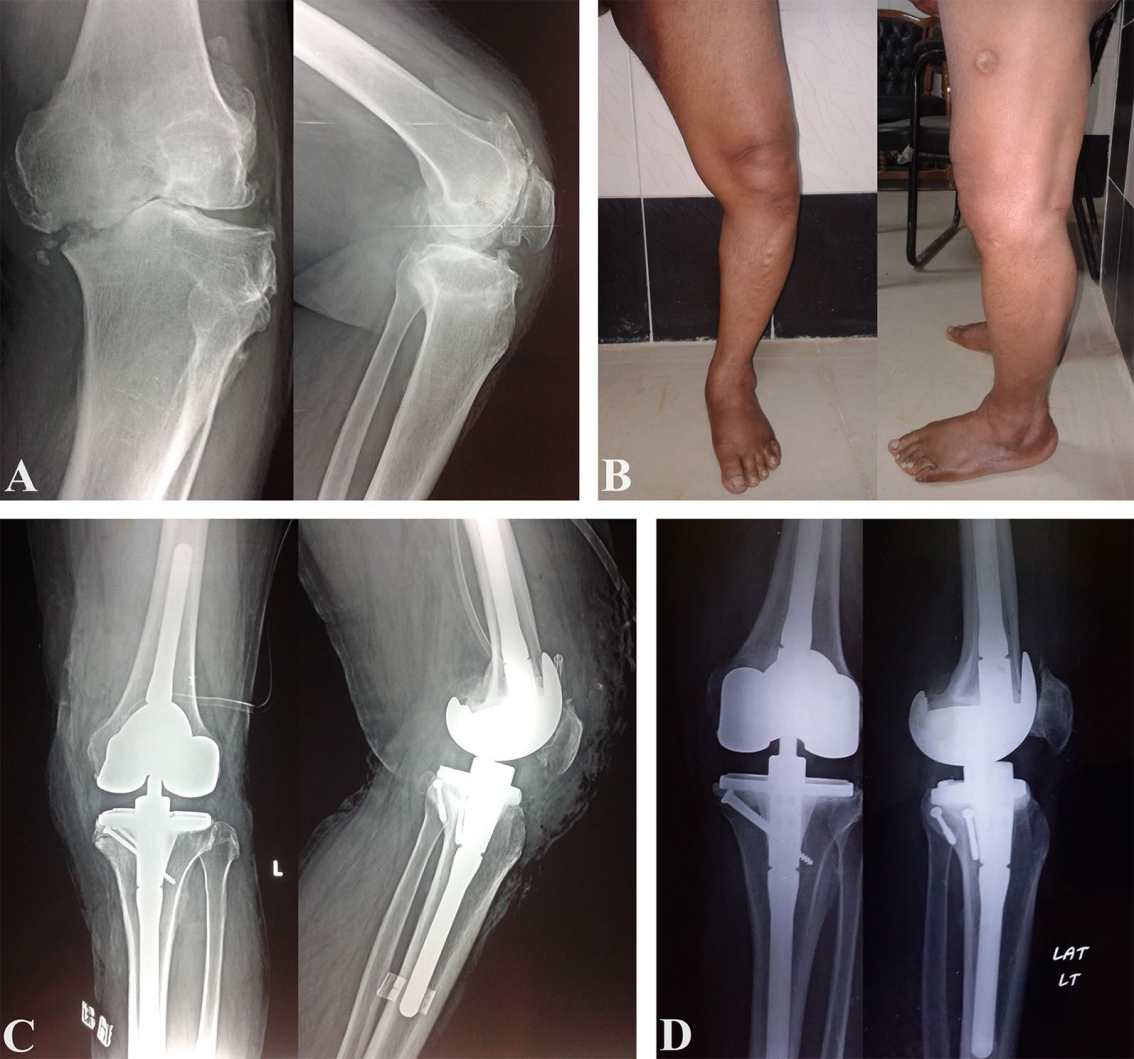
A 58-year-old male with advanced left knee OA. **A** Preoperative X-rays showing advanced OA and the medial proximal tibial defect. **B** Preoperative standing photographs showing varus deformity. **C** Postoperative X-rays after TKA using LCCK and reconstruction of the defect using structural autograft block fixed with two screws. **D** Three-year follow-up X-rays showing complete union of the graft

Patients were followed up at 6 weeks, 3 months, and 6 months, then yearly, and were evaluated clinically with Knee Society Score (KSS) [18] and Western Ontario and McMaster Universities Osteoarthritis Index (WOMAC) [19]. Follow-up knee X-rays were also done at 6 weeks, 3 months, 6 months, and then yearly to assess component stability and bone graft union, Fig. 3.

### Statistical analysis

Data were analyzed using IBM SPSS software package version 20.0. (Armonk, NY: IBM Corp).

Qualitative data were expressed as numbers and percentages. Quantitative data were expressed as mean, and standard deviation (SD) when normally distributed and as median and interquartile range (IQR) when not normally distributed.

Comparison of preoperative and postoperative continuous data was done using the paired samples *t*-test or the Wilcoxon signed-rank test when appropriate. Comparison of the effect of different variables on the functional and radiological outcomes was done using the Student’s *t*-test, the Mann–Whitney *U*-test, the one-way ANOVA, or the Kruskal–Wallis *H*-test, when applicable. Significance of the obtained results was judged at the 5% level.

## Results

### Demographics and baseline characteristics

This study included 48 patients, including 25 (52.1%) patients < 70 years and 23 (47.9%) patients ≥ 70 years, with a mean age of 69.2 ± 4.5 (range, 58–78) years. Among the participants, 18 (37.5%) were males and 30 (62.5%) were females. The mean BMI was 31.4 ± 3.7 (range, 25.4–41.5) kg/m^2^. Regarding the BMI classification, 21 (43.8%) patients had obesity class I, and 19 (39.6%) patients were overweight.

TKA was done on the left knee in 27 (56.3%) patients and the right knee in 21 (43.8%) patients.

Regarding the preoperative aFTA, 31 (64.6%) patients had a 10–25° varus angulation, while 17 (35.4%) patients had > 25° varus angulation. Additionally, 22 (45.8%) patients had a flexion contracture of > 10°, Table 1.

**Table 1:** Demographics and baseline characteristics of the included patients

Characteristics	Value (*n* = 48)
Age, years (*n*, %)	
< 70	25 (52.1%)
≥ 70	23 (47.9%)
Age, years (mean ± SD)	69.2 ± 4.5
Gender (*n*, %)	
Male	18 (37.5%)
Female	30 (62.5%)
BMI, kg/m^2^ (mean ± SD)	31.4 ± 3.7
BMI classification (*n*, %)	
Overweight (25–29.9)	19 (39.6%)
Obesity class I (30–34.9)	21 (43.8%)
Obesity class II (35–39.9)	7 (14.6%)
Obesity class III (≥ 40)	1 (2.1%)
Side of arthroplasty (*n*, %)	
Right	21 (43.8%)
Left	27 (56.3%)
Preoperative aFTA (*n*, %)	
10–25° varus	31 (64.6%)
> 25° varus	17 (35.4%)
Preoperative flexion contracture (*n*, %)	
≤ 10°	26 (54.2%)
> 10°	22 (45.8%)

*SD* standard deviation, *BMI* body mass index, *aFTA* anatomical femorotibial angle

The mean depth of defect as measured intraoperatively was 17 ± 3.6 (range, 10–20) mm. The mean thickness of the used polyethylene insert was 13 ± 1.7 (range, 10–16) mm. The average operative time was 129.3 ± 6.7 (range, 120–140) minutes. Primary TKA prosthesis with stemmed tibial component was used in 38 (79.2%) patients, and LCCK prosthesis was used in 10 (20.8%) patients with severe lateral ligament laxity.

### Functional and radiological outcomes

The average follow-up period was 52.2 ± 12.3 (range, 36–96) months. The median KSS improved significantly from 30 (IQR, 27–35) preoperatively to 89 (IQR, 85–93) at the last follow-up, *P* < 0.001. Additionally, there was a significant reduction in the median WOMAC score from 85 (IQR, 80–90) preoperatively to 30.5 (IQR, 27–35) at last follow-up, *P* < 0.001.

The mean flexion–extension ROM increased significantly from 73 ± 12.4 (range, 45–100) degrees preoperatively to 124 ± 8.4 (range, 95–135) degrees at the last follow-up, *P* < 0.001. In addition, there was significant correction of the varus malalignment and the flexion contractures, *P* < 0.001, Table 2. The mean graft union time was 4.9 ± 1 (range, 3–8) months.

**Table 2:** Comparison between preoperative and postoperative parameters

	Preoperative	Last follow-up	*P*-value*
KSS			
Median (IQR)	30 (27–35)	89 (85–93)	< 0.001
WOMAC			
Median (IQR)	85 (80–90)	30.5 (27–35)	< 0.001
Range of motion, °			
Mean ± SD	73 ± 12.4	124 ± 8.4	< 0.001
aFTA, °			
Median (IQR)	25 (25–30)	0 (0–0)	< 0.001
Flexion contracture, °			
Median (IQR)	10 (7.5–15)	0 (0–0)	< 0.001

*KSS* Knee Society Score, *IQR* interquartile range, *WOMAC* Western Ontario and McMaster Universities Arthritis Index, *SD* standard deviation, *aFTA* anatomical femorotibial angle

^*^ Statistically significant at *P* ≤ 0.05

### Factors affecting the outcomes

At the last follow-up, patients younger than 70 years had a significantly higher median KSS score than those ≥ 70 years, 90 (IQR, 85–93) and 85 (IQR, 83–90), respectively, *P* = 0.016. Additionally, the score was higher in overweight patients, 90 (IQR, 87–93), compared with patients with obesity class I, 88 (IQR, 85–90) and obesity class II, 85 (IQR, 85–89), *P* = 0.049, Table 3.

**Table 3:** Relation between preoperative variables and the last follow-up Knee Society Score (KSS)

Preoperative variables	Last follow-up KSS	*P*-value*
	Median (IQR)	
Age		
< 70 years	90 (85–93)	**0.016**
≥ 70 years	85 (83–90)	
Gender		
Male	89 (85–93)	0.870
Female	89 (85–93)	
BMI classification		
Overweight (25–29.9)	90 (87–93)	**0.049**
Obesity class I (30–34.9)	88 (85–90)	
Obesity class II (35–39.9)	85 (85–89)	
Obesity class III (≥ 40)^#^	–	
Side of arthroplasty		
Right	89 (85–93)	0.882
Left	89 (85–90)	
Preoperative aFTA		
10–25° varus	89 (85–93)	0.189
> 25° varus	85 (85–90)	
Preoperative flexion contracture		
≤ 10°	86.5 (85–90)	0.320
> 10°	89 (85–93)	

*IQR*, interquartile range; *BMI*, body mass index; *aFTA*, anatomical femorotibial angle

^*^ Statistically significant at *P* ≤ 0.05

^#^ Excluded from the comparison due to small number of cases (*n* = 1)

Regarding the last follow-up WOMAC, patients with obesity class II had a significantly higher median WOMAC, 40 (IQR, 37.5–40.2), compared with overweight patients, 27.3 (IQR, 27–33, and patients with obesity class I, 31 (IQR, 27–35), *P* = 0.007, Table 4.

**Table 4:** Relation between preoperative variables and the last follow-up Western Ontario and McMaster Universities Arthritis Index (WOMAC)

Preoperative variables	Last follow-up WOMAC	*P*-value*
	Median (IQR)	
Age		
< 70 years	28 (27–35)	0.186
≥ 70 years	35 (27.3–35)	
Gender		
Male	30 (27–35)	0.621
Female	31 (27–38)	
BMI classification		
Overweight (25–29.9)	27.3 (27–33)	**0.007**
Obesity class I (30–34.9)	31 (27–35)	
Obesity class II (35–39.9)	40 (37.5–40.2)	
Obesity class III (≥ 40)^#^	–	
Side of arthroplasty		
Right	28 (27–35)	0.801
Left	31 (27.2–35)	
Preoperative aFTA		
10–25° varus	30 (27–35)	0.836
> 25° varus	33 (27–35)	
Preoperative flexion contracture		
≤ 10°	33 (27.3–38)	0.120
> 10°	28 (26–35)	

*IQR* interquartile range, *BMI* body mass index, *aFTA* anatomical femorotibial angle

^*^ Statistically significant at *P* ≤ 0.05

^#^ Excluded from the comparison due to small number of cases (*n* = 1)

At the last follow-up, males had a significantly higher mean flexion–extension ROM compared with females, 128.3 ± 5.9 (range, 110–135) degrees and 121.3 ± 8.7 (range, 95–130), *P* = 0.004. Overweight patients had a significantly higher mean ROM, 127.4 ± 4 (range, 120–135), than patients with obesity class I, 124 ± 7.8 (range, 110–135) and obesity class II, 118.6 ± 8.5 (range, 105–130) degrees, *P* = 0.020, Table 5.

**Table 5:** Relation between preoperative variables and the last follow-up flexion–extension range of motion (ROM)

Preoperative variables	Last follow-up ROM	*P*-value*
	Mean ± SD	
Age		
< 70 years	124.2 ± 8.1	0.839
≥ 70 years	123.7 ± 8.9	
Gender		
Male	128.3 ± 5.9	**0.004**
Female	121.3 ± 8.7	
BMI classification		
Overweight (25–29.9)	127.4 ± 4.8	**0.020**
Obesity class I (30–34.9)	124 ± 7.8	
Obesity class II (35–39.9)	118.6 ± 8.5	
Obesity class III (≥ 40)^#^	95	
Side of arthroplasty		
Right	124 ± 9.3	0.949
Left	123.9 ± 7.9	
Preoperative aFTA		
10–25° varus	123.1 ± 8.9	0.327
> 25° varus	125.6 ± 7.5	
Preoperative flexion contracture		
≤ 10°	122.5 ± 9.6	0.183
> 10°	125.7 ± 6.6	

*SD* standard deviation, *BMI* body mass index, *aFTA* anatomical femorotibial angle

^*^ Statistically significant at *P* ≤ 0.05

^#^ Excluded from the comparison due to small number of cases (*n* = 1)

The mean graft union time was higher in patients ≥ 70 years, 5.3 ± 1.0 (range, 4–8), compared with patients < 70 years, 4.5 ± 0.8 (range, 3–6) months, *P* = 0.002, Table 6.

**Table 6:** Relation between preoperative variables and the graft union time

Preoperative variables	Graft union time(months)	*P*-value*
	Mean ± SD	
**Age**		
< 70 years	4.5 ± 0.8	**0.002**
≥ 70 years	5.3 ± 1.0	
**Gender**		
Male	4.7 ± 1.1	0.322
Female	5.0 ± 0.9	
**BMI classification**		
Overweight (25–29.9)	5.1 ± 1.3	0.451
Obesity class I (30–34.9)	4.9 ± 0.7	
Obesity class II (35–39.9)	4.5 ± 0.4	
Obesity class III (≥ 40)^**#**^	6	
**Side of arthroplasty**		
Right	4.7 ± 0.8	0.303
Left	5.0 ± 1.1	
**Preoperative aFTA**		
10–25° varus	4.8 ± 0.9	0.434
> 25° varus	5.1 ± 1.2	
**Preoperative flexion contracture**		
≤ 10°	5.0 ± 1.1	0.678
> 10°	4.8 ± 0.9	

*SD* standard deviation, *BMI* body mass index, *aFTA* anatomical femorotibial angle

^*^ Statistically significant at *P* ≤ 0.05

^#^ Excluded from the comparison due to small number of cases (*n* = 1)

### Complications

Persistent medial side joint pain occurred in two (4.2%) patients, most probably due to pes anserine bursitis. Additionally, two (4.2%) patients had delayed graft union at 7 and 8 months.

## Discussion

Patients with advanced knee OA and varus deformity usually have uncontained proximal tibial bone defects, which are a technically demanding aspect of primary TKA [20]. Achieving successful outcomes relies on properly positioning and aligning the implant components [21].

In this study, uncontained medial tibial bone defects were managed by autograft reconstruction, taken from the distal femur cuts. With a mean follow-up of 52.2 months, there was a significant improvement in the KSS and WOMAC scores and the flexion–extension ROM, with significant correction of the varus malalignment and the flexion contracture. Better functional scores were observed in patients < 70 years and patients with lower BMI. A better range of motion was observed in male patients and patients with lower BMI. Graft union was faster in patients < 70 years.

Autogenous structural bone grafts have been described  as a treatment option for uncontained tibial bone defects of 5–10 mm in diameter, with good long-term outcomes [22].

In our study, significant improvement of WOMAC scores was achieved in the form of reduction from 85 preoperatively to 30.5, with an average follow-up of 52.2 months. Additionally, the median KSS improved significantly from 30 before surgery to 89 at last follow-up.

Chon et al. [13] used autogenous structural and cancellous chip bone graft for the reconstruction of medial proximal tibial defects of 10 mm or more in depth in 40 patients undergoing primary TKA. With At least 1 year of follow-up, a significant improvement in WOMAC scores was achieved [13]. Yoon et al. [10] reported the outcomes of using autogenous onlay bone graft in 19 patients (22 knees) with an average 12-mm-deep uncontained medial tibial bone defects undergoing primary TKA. With a mean follow-up period of 30.2 months, there was a significant increase in the mean KSS score from 30 preoperatively to 92 at the last follow-up [10].

In our study, significant improvement of knee ROM was achieved. Similarly, Chon et al. [13] reported significant improvement in the ROM.

In our study, bony union was achieved in all cases at final follow-up, with a mean graft union time of 4.9 ± 1 months. Similarly, Chon et al. [13] reported that all cases showed bone union at the graft-bone interface. Yoon et al. [10] reported a mean time of 3.2 months for solid union. Kharbanda and Sharma [23] reported an average graft incorporation time of 4.5 months in 54 knees in their study of autograft reconstructions for bone defects with an average follow-up of 7.8 years.

In the current study, no significant complications were reported, such as infection, implant loosening or graft nonunion. Revision surgery was not required in any patients. Similarly, no significant complications were reported in Chon et al. [13] and Yoon et al. [10] studies.

In the standard tibial component of TKA, 53–67% of load sharing occurs at the cortical rim [24]. Thus, uncontained defects without cortical support must be addressed adequately to ensure prosthesis stability [25].

In general, it is recommended to use bone grafting for defects of 5–10 mm, while metal augmentation has been described to address these uncontained defects, especially for defects exceeding 10 mm [26–28]. Nevertheless, achieving anatomical bone reconstruction is not feasible with metal augments, necessitating unavoidable extra bone resection to allow a proper fit [29]. Additionally, persistent knee pain may result from augment protrusion [12]. Metal blocks also result in substantial bone defects in subsequent revision surgeries [11].

The use of structural allografts has also been described [9]. However, several drawbacks exist, including graft nonunion, collapse or resorption, in addition to the risk of disease transmission such as human immunodeficiency virus (HIV), hepatitis C virus (HCV), and human T-lymphotropic virus (HTLV) [30, 31]. Iwase et al. [9] reported a case of nonunion and another case with radiolucent line out of 17 patients treated with allogenous structural bone graft.

Autogenous bone grafting has the advantage of providing biological stability while reinforcing the bone stock and lowering medical costs [32].

Using structural autogenous bone grafting for uncontained bone defects ≥ 10 mm deep, we have obtained good outcomes without significant complications such as infection, graft nonunion, graft resorption, or implant loosening. Using local autograft blocks from the knee cuts avoids donor site morbidity in other anatomical areas of the skeleton.

A stemmed tibial component and stable graft fixation with screws are paramount to ensure the stability of the prosthesis. Watanabe et al. [32] reported a 100% autograft union rate in 30 patients without using screws. However, using cancellous screws for fixing autologous bone grafts allows robust initial fixation, therefore achieving a high rate of bony union and implant stability [33].

The current study has limitations, such as the lack of control group. Preoperative computed tomography (CT) scans were not done to measure volumetric loss. Follow-up CT scans were not done to assess the cross-trabeculation between the graft and the proximal tibial bone. Additionally, we measured only the depth of the defect, with the inability to measure the volume of the defect intraoperatively. The defects have variable sizes and shapes, and there should be a specific device that is able to measure the volume intraoperatively.

Future comparative studies between structural autogenous bone grafts and other options, such as structural allografts or metal augments should be conducted to further recommend the ideal method of reconstruction of uncontained tibial defects with a depth ≥ 10 mm. Also, a long-term follow-up study is needed to assess the potential complications associated with screw fixation and its impact on the stability and durability of the implant.

## Conclusions

Uncontained medial proximal tibial defects ≥ 10 mm deep in patients undergoing primary TKA can be adequately managed using structural autogenous bone graft fixed with screws. This technique has satisfactory clinical and radiological outcomes without significant complications.

## Data Availability

The dataset analyzed in this study is available from the corresponding author on request.
